# Behavioral impairment in SHATI/NAT8L knockout mice *via* dysfunction of myelination development

**DOI:** 10.1038/s41598-017-17151-1

**Published:** 2017-12-04

**Authors:** Kazuyuki Sumi, Kyosuke Uno, Hiroshi Noike, Takenori Tomohiro, Yasumaru Hatanaka, Yoko Furukawa-Hibi, Toshitaka Nabeshima, Yoshiaki Miyamoto, Atsumi Nitta

**Affiliations:** 1 Department of Pharmaceutical Therapy and Neuropharmacology, Faculty of Pharmaceutical Sciences, Graduate School of Medicine and Pharmaceutical Sciences, University of Toyama, Toyama, 930-0194 Japan; 2 Laboratory of Biorecognition Chemistry, Graduate School of Medicine and Pharmaceutical Sciences, University of Toyama, Toyama, 930-0194 Japan; 3 Department of Neuropsychopharmacology and Hospital Pharmacy, Nagoya University Graduate School of Medicine, Nagoya, 466-8560 Japan; 4 Advanced Diagnostic System Research Laboratory, Fujita Health University, Graduate School of Health Sciences, Toyoake, 470-1192 Japan; 5 Aino Universityy, Ibaragi, 567-0012 Japan

## Abstract

We have identified SHATI/NAT8L in the brain of mice treated with methamphetamine. Recently, it has been reported that SHATI is *N*-acetyltransferase 8-like protein (NAT8L) that produces *N*-acetylaspatate (NAA) from aspartate and acetyl-CoA. We have generated SHATI/NAT8L knockout (*Shati* ^−/−^) mouse which demonstrates behavioral deficits that are not rescued by single NAA supplementation, although the reason for which is still not clarified. It is possible that the developmental impairment results from deletion of SHATI/NAT8L in the mouse brain, because NAA is involved in myelination through lipid synthesis in oligodendrocytes. However, it remains unclear whether SHATI/NAT8L is involved in brain development. In this study, we found that the expression of *Shati/Nat8l* mRNA was increased with brain development in mice, while there was a reduction in the myelin basic protein (MBP) level in the prefrontal cortex of juvenile, but not adult, *Shati* ^−/−^ mice. Next, we found that deletion of SHATI/NAT8L induces several behavioral deficits in mice, and that glyceryltriacetate (GTA) treatment ameliorates the behavioral impairments and normalizes the reduced protein level of MBP in juvenile *Shati* ^−/−^ mice. These findings suggest that SHATI/NAT8L is involved in myelination in the juvenile mouse brain via supplementation of acetate derived from NAA. Thus, reduction of SHATI/NAT8L induces developmental neuronal dysfunction.

## Introduction

SHATI has been identified as a novel molecule from the nucleus accumbens (NAc) of mice treated with methamphetamine^1^. It was reported that SHATI is *N*-acetyltransferase 8-like protein (NAT8L) that produces *N*-acetylaspatate (NAA) from aspartate and acetyl-CoA^2,3^. Here, we describe SHATI/NAT8L instead of SHATI.

Magnetic resonance spectroscopy of the human brain shows a large amount of NAA signal. Therefore NAA is often used as a putative neuronal marker. Moreover, it has been reported that NAA is decreased in psychiatric disorders such as schizophrenia, attention deficit hyperactivity disorder, and drug dependence^4–6^. NAA is used for the production of *N*-acetylaspartylglutamate (NAAG) in neuronal cells in mammals, and NAAG is a highly selective endogenous metabotropic glutamate receptor (mGluR) 3 agonist^7,8^. Previously, we have reported that overexpression of SHATI/NAT8L in the NAc of mice attenuates the response to METH through the mGluR3 signaling activated by NAAG^9^. Furthermore, NAA is metabolized to aspartate and acetate by aspartoacylase (ASPA) in oligodendrocytes in the brain. Then acetate is converted to acetyl-CoA and used for lipid synthesis and myelination^10^. Moreover, it has reported that deletion of ASPA in the mice results in impaired postnatal myelination and this mouse is used for the model of Canavan disease, defects in NAA metabolism^11^. These reports suggest that SHATI/NAT8L has multiple roles in the central nervous system via the synthesis of NAA.

Recently, it was reported that SHATI/NAT8L knockout (*Shati* ^−/−^) mice show decreased NAA content in the brain, reduced social interaction and shortened immobility time in the forced swimming test^12^. Moreover, it was also reported that the decreased level of brain-derived neurotrophic factor (BDNF) mRNA in the prefrontal cortex of *Shati* ^−/−^ mice^13^. Importantly, single injection of NAA into ventricles could not completely improve behavioral deficits of *Shati* ^−/−^ mice, although the impairment in *Shati* ^+/−^ mice was ameliorated by the same treatment with NAA. It is possible that the behavioral deficits caused by complete deletion of SHATI/NAT8L are related to the developmental impairment, because NAA is involved in myelination through lipid synthesis in oligodendrocytes, although the number of neuronal cell are not changed in *Shati* ^−/−^ mice^14,15^. In the brain, neuron-glia communication plays regulatory roles in the central nervous system functions^16^. In particular, myelin supports neuronal signaling but dysfunction of myelin induces reduced social interaction and other behavioral deficits in mice^17,18^. However, it remains unclear whether SHATI/NAT8L is involved in the development of the brain especially, in myelination. There are several reports that impaired differentiation of myelination and oligodendrocytes in the prefrontal cortex induces depressive social behaviors, and that the prefrontal cortex has been proposed to be an important brain region for social interaction in mice^18,19^.

In the present study, we investigated the change in the expression of *Shati/Nat8L* mRNA in developing brain, and found that deletion of SHATI/NAT8L altered the myelin basic protein (MBP) level in the prefrontal cortex of juvenile, but not adult, mice. These findings suggest that SHATI/NAT8L is involved in brain development. Next, we demonstrated that deletion of SHATI/NAT8L induces several behavioral deficits such as hyperactivity, reduction of social interaction, and induction of impulsiveness. Moreover, glyceryltriacetate (GTA), a supply of acetate, treatment during the juvenile period ameliorates hyperactivity, reduced social interaction and impulsiveness caused by deletion of SHATI/NAT8L. Furthermore, reduced level of MBP in juvenile *Shati* ^−/−^ mice was normalized by GTA treatment. These results suggest that SHATI/NAT8L might be involved in myelination in the juvenile mice brain via supplementation of acetate derived from NAA, and that the hyperactivity, social deficits and impulsiveness in *Shati* ^−/−^ mice are induced by NAA deficit. Taken together with our new findings, NAA and/or SHATI/NAT8L are required for myelination in the developing brain of mice, and their deficit could induce behavioral deficits. These factors could provide new targets for the treatment of psychiatric disorders, such as attention deficit hyperactivity disorder.

## Results

### Expression of Shati/Nat8l mRNA increases with brain development in mice

**Figure 1:**
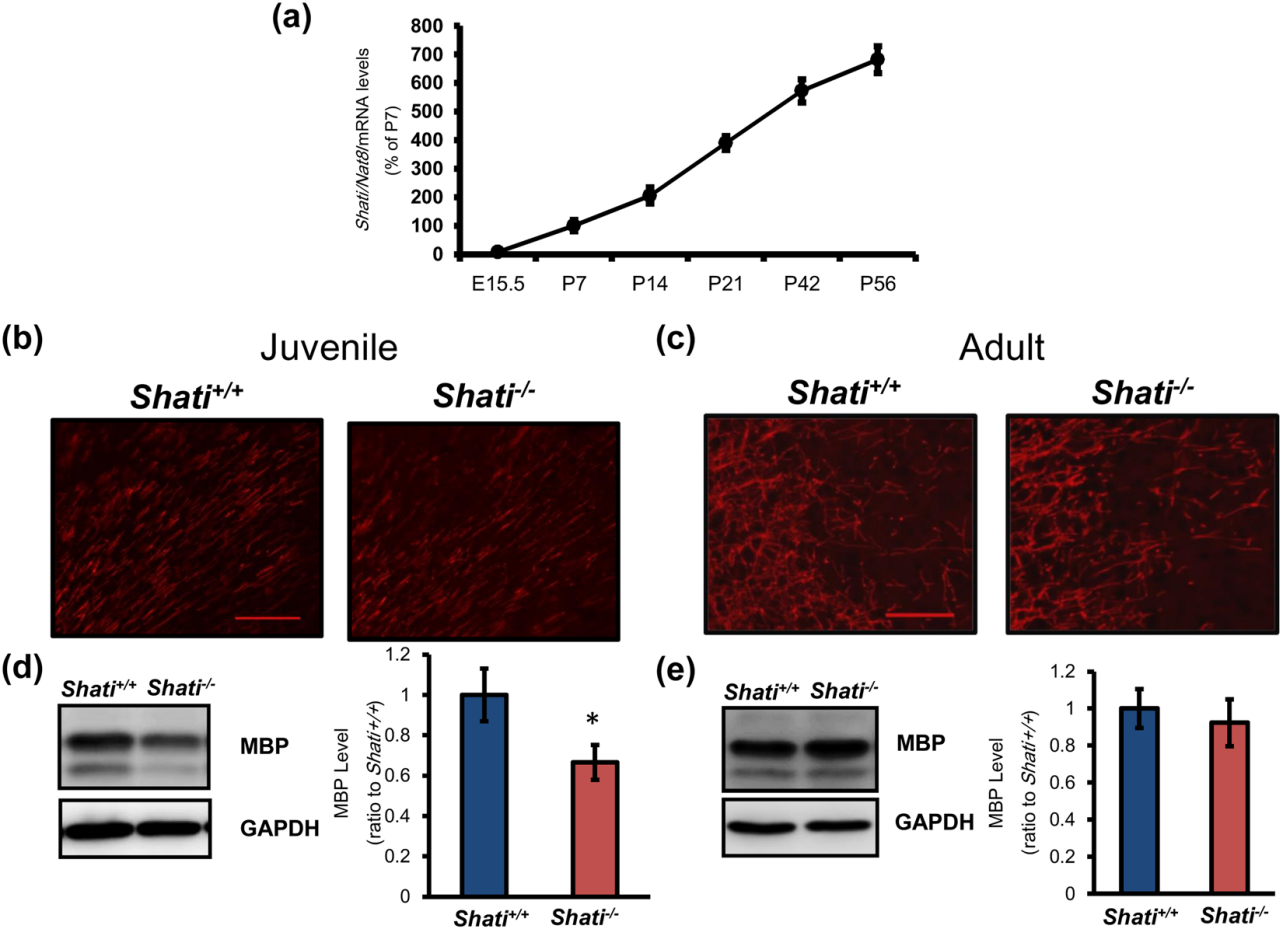
Expression of *Shati/Nat8l* mRNA was increased depending on the brain development and SHATI/NAT8L affects the expression of MBP. (**a**) Real-time RT-PCR analysis of *Shati/Nat8l* mRNA in whole brains of mice was performed. To standardize the PCR products, we used primers for *36B4* as an internal control. The *Shati/Nat8l* mRNA levels were expressed as the percentage relative to P7 expression. Values represent the mean ± S.E.M. (n = 3). (**e**) embryonic day, P: postnatal day. (**b**,**c**) The expression pattern of MBP in juvenile (3 weeks old) and adult (10 weeks old) *Shati* ^+/+^ and *Shati* ^−/−^ mice was detected by immunohistochemistry analysis. Scale bars in the figure = 100 μm (**d**,**e**) The expression pattern of MBP in between juvenile and adult *Shati* ^+/+^ and *Shati* ^−/−^ mice was detected by Western blot analysis. Values represent the mean ± S.E.M. (n = 5). *p < 0.05 vs. *Shati* ^+/+^ mice (Student’s t test). Full-length blots are presented in Supplemental Figs S2 and 3.

We collected the whole brain for the measurement of *Shati/Nat8l* mRNA during brain development, because the brain development began from embryonic state and *Shati/Nat8l* mRNA is expressed in whole brain. To analyze the expression of *Shati/Nat8l*, we first quantified *Shati/Nat8l* mRNA levels in the whole brain on various days at the age of embryonic (E) 15.5, postnatal (P) 7, 14, 21, 42 and 56. *Shati/Nat8l* mRNA is strongly expressed only after birth, while *Shati/Nat8l* mRNA expression level during the embryonic stage and juvenile stage were very low (Fig. 1a; E15.5 = 8.72 ± 1.39%, P7 = 100.00 ± 19.67%, P14 = 205.78 ± 28.58%, P21 = 390.11 ± 24.09%, P42 = 572.32 ± 40.62% and P56 = 681.96 ± 46.70%; Ct value of 36B4: E15.5 = 31.8 ± 0.3, P7 = 31.7 ± 0.5, P14 = 31.2 ± 0.3, P21 = 31.7 ± 0.1, P42 = 31.9 ± 0.2, P56 = 32.0 ± 0.2; Ct value of *Shati*: E15.5 = 38.4 ± 0.1, P7 = 36.2 ± 0.3, P14 = 34.6 ± 0.1, P21 = 34.2 ± 0.1, P42 = 33.8 ± 0.2, P56 = 33.3 ± 0.3). These findings indicate that expression of *Shati/Nat8l* mRNA increases with whole brain development in mice.

### Deletion of Shati/Nat8l altered the MBP level in the brain of juvenile, but not adult mice

We next investigated whether the deletion of SHATI/NAT8L affected the myelin basic protein (MBP) level in the prefrontal cortex, which is involved in the myelination and emotional behaviors in mice^17,18^. To account for several isoforms of MBP, we detected protein bands from 10 kDa to 25 kDa in the Western blot analysis as previous report^20^. Immunohistochemical analysis and Western blotting showed the expression of MBP in the prefrontal cortex of juvenile (3 weeks old) mice was decreased in *Shati* ^−/−^ mice compared with that of *Shati* ^+/+^ mice (Fig. 1b and d; *Shati* ^+/+^ mice = 1.00 ± 0.13, *Shati* ^−/−^ mice = 0.66 ± 0.09, t_4_ = 4.754, *p* < 0.05). On the other hand, the expression of MBP in the prefrontal cortex of the adult (10 weeks old) mice was unchanged in *Shati* ^−/−^ mice (Fig. 1c,e; *Shati* ^+/+^ mice = 1.00 ± 0.10, *Shati* ^−/−^ mice = 0.92 ± 0.13, t_4_ = 2.483, n.s.). These findings indicate that SHATI/NAT8L plays an important role in the regulation of myelin state in the brain of juvenile mice.

### GTA treatment ameliorated deficit of social interaction caused by deletion of SHATI/NAT8L in mice

**Figure 2:**
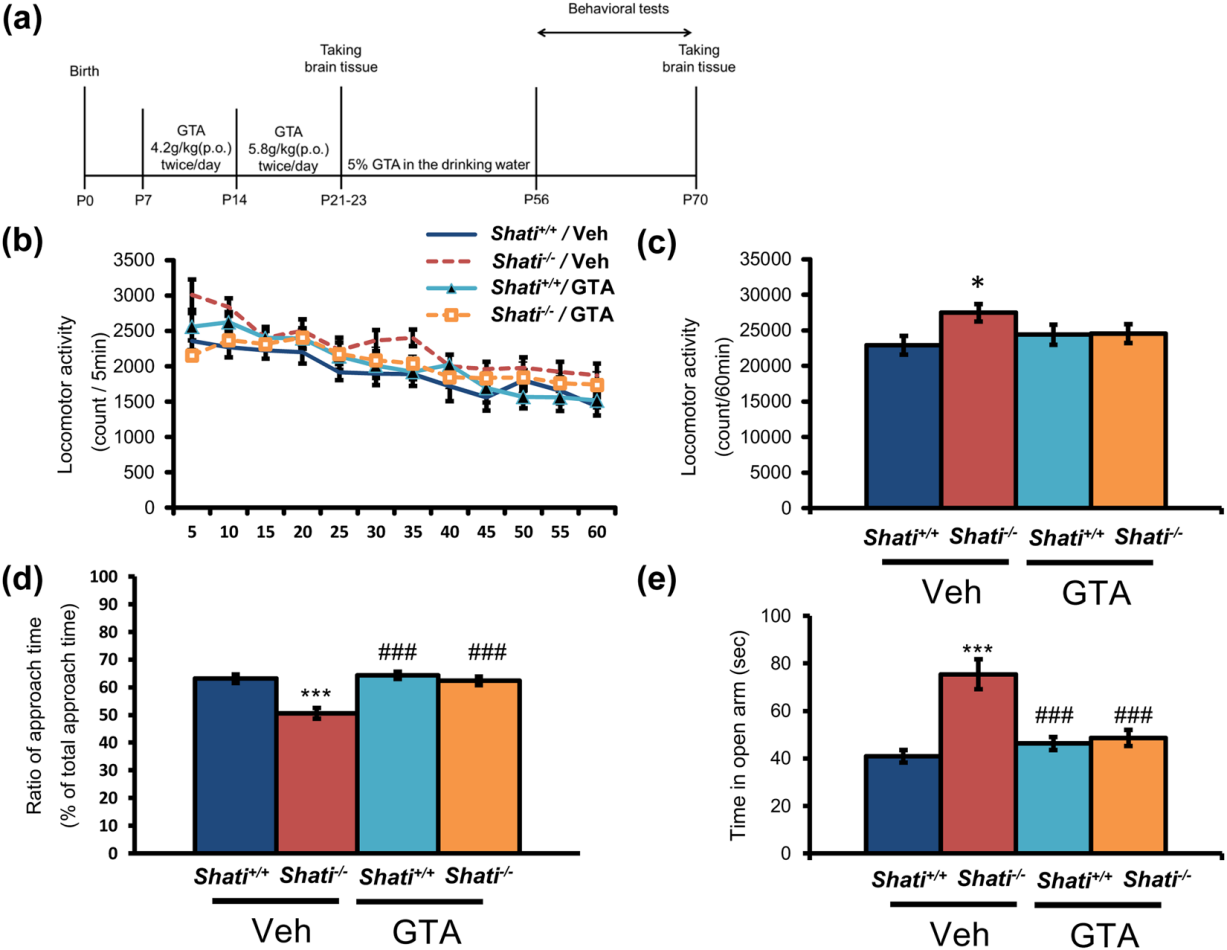
GTA treatment ameliorates reduced behavioral deficits caused by the deletion of SHATI/NAT8L. (**a**) GTA treatment and several behavioral experiments were performed following this schedule. After GTA treatment, behavioral experiments were performed in the following order; locomotor activity, social interaction test and elevated plus-maze test. (**b**,**c**) A difference in basal locomotor activity was observed in a novel environment between *Shati* ^+/+^ and *Shati* ^−/−^ mice. On the other hand, GTA itself treatment did not affect the locomotor activity in *Shati* ^+/+^ mice. Veh: vehicle, GTA: glyceryltriacetate. Values represent the mean ± SEM. (n = 10). *p < 0.05 vs. *Shati* ^+/+^/Veh mice (ANOVA followed by Bonferroni’s post-hoc test). (**d**) In the three-chambered social interaction test, *Shati* ^+/+^ mice were more interested in a stranger mouse compared with *Shati* ^*−/−*^ mice. Values represent the mean ± SEM. (n = 12). ***p < 0.001 vs. *Shati* ^+/+^/Veh mice, ^###^ *p* < 0.001 vs. *Shati* ^−/−^/Veh mice (ANOVA followed by Bonferroni’s post-hoc test) (**e**) A difference in the duration of time spent in the open arms of the elevated plus-maze test was observed, indicating a difference in anxiety-like behavior between *Shati* ^−/−^ and *Shati* ^+/+^ mice. Values represent the mean ± S.E.M. (n = 12). ***p < 0.001 vs. *Shati* ^+/+^/Veh mice, ^###^ *p* < 0.001 vs. *Shati* ^−/−^/Veh mice (ANOVA followed by Bonferroni’s post-hoc test). Other data of behavioral experiments are represented in Supplemental Fig. S1.

**Figure 3:**
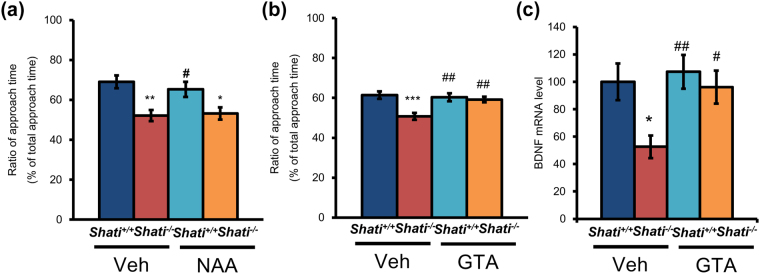
Behavioral deficit in *Shati* ^−/−^ mice was not recovered by NAA repeated administration. (**a**) In three-chambered social interaction test, the mice were administrated NAA into the cerebral ventricle each test days. *Shati* ^−/−^ mice were more interested to a novel object, but not to a stranger mouse compared with *Shati* ^+/+^ mice. NAA repeated administration did not ameliorates reduced behavioral deficits in *Shati* ^−/−^ mice. Values represent the mean ± SEM. (n = 8). **p < 0.01, *p < 0.05 vs. *Shati* ^+/+^/Veh mice, ^#^ *p* < 0.05 vs. *Shati* ^−/−^/Veh mice (ANOVA followed by Bonferroni’s post-hoc test) (**b**) GTA was treated orally to *Shati* ^+/+^ and *Shati* ^−/−^ pups from postnatal day 7 to 21. *Shati* ^−/−^ mice showed reduced social interaction, but it was ameliorated by juvenile GTA treatment. Values represent the mean ± SEM. (n = 8). ***p < 0.001 vs. *Shati* ^+/+^/Veh mice, ^##^ *p* < 0.01 vs. *Shati* ^−/−^/Veh mice (ANOVA followed by Bonferroni’s post-hoc test) (**c**) *BDNF* mRNA levels were measured by Real-time RT-PCR analysis. The difference in the level of BDNF mRNA between *Shati* ^+/+^ and *Shati* ^−/−^ mice was detected, but GTA treatment rescue these difference. Values represent the mean ± S.E.M. (n = 8) *p < 0.05 vs. *Shati* ^+/+^/Veh mice, ^#^ *p* < 0.05, ^##^ *p* < 0.01 vs. *Shati* ^−/−^/Veh mice (ANOVA followed by Bonferroni’s post-hoc test).

We investigated whether the behavioral deficits observed in *Shati* ^−/−^ mice^12,13^ were ameliorated by acetate supplementation, because NAA which synthesized by SHATI/NAT8L is metabolized to aspartate and acetate by aspartoacylase in oligodendrocytes. For acetate supplementation, we used GTA which metabolized to acetate and distributed to the brain rapidly after oral administration as previous reports^21,22^. Firstly, we assessed the validity of behavioral test in *Shati* ^−/−^ mice (Supplemental Fig. 1). In the locomotor activity test to check the change of spontaneous movement, *Shati* ^−/−^ mice showed higher activity than *Shati* ^+/+^ mice (Supplemental Fig. 1A,B; *Shati* ^+/+^ mice = 23132.88 ± 981.77 counts, *Shati* ^−/−^ mice = 26662.53 ± 1000.57 counts, t_14_ = 2.486, *p* < 0.05). Next, we reconfirmed the memory by the deletion of SHATI/NAT8L. In the Y-maze test, there was no difference in spontaneous alternation between *Shati* ^+/+^ and *Shati* ^−/−^ mice (Supplemental Fig. 1C; *Shati* ^+/+^ mice = 71.06 ± 1.74%, *Shati* ^−/−^ mice = 69.18 ± 2.80%, t_8_ = 0.3683, n.s.). Furthermore, in the three-chambered social interaction test to investigate the social behavior, *Shati* ^−/−^ mice showed reduced social interaction to a stranger mice compared with *Shati* ^+/+^ mice while it showed increased interaction to object compared with Shati^−/−^ mice (Supplemental Fig. 1D; Stranger; *Shati* ^+/+^ mice = 63.63 ± 1.78%, *Shati* ^−/−^ mice = 49.80 ± 2.54%, t_8_ = 4.599, *p* < 0.001). Moreover, in the elevated plus maze test to test impulsivity, *Shati* ^−/−^ mice spent longer in the open arms compared with *Shati* ^+/+^ mice (Supplemental Fig. 1E; *Shati* ^+/+^ mice = 34.97 ± 2.63 s, *Shati* ^−/−^ mice = 81.80 ± 8.36 s, t_8_ = 5.463, *p* < 0.001). Taken together, *Shati* ^−/−^ mice showed hyper locomotion, impulsivity and social deficits. These results are agreed with our previous publications^12,13^. We treated mice with GTA or vehicle (Veh) from the age of P7 to the age of 8 weeks old (Fig. 2a). After GTA treatment, behavioral experiments were performed in the following order; locomotor activity, social interaction test and elevated plus-maze test. GTA normalized locomotor activity of *Shati* ^−/−^ mice as the level of *Shati* ^+/+^ mice groups, although *Shati* ^−/−^ mice showed higher locomotor activity compared with *Shati* ^+/+^ mice (Fig. 2b,c; *Shati* ^+/+^/Veh mice = 22917.3 ± 1359.6 counts, *Shati* ^−/−^/Veh mice = 26761.3 ± 999.6 counts, *Shati* ^+/+^/GTA mice = 24406.2 ± 1419.8 counts, *Shati* ^−/−^/GTA mice = 24556.6 ± 1302.8 counts; *Shati* ^+/+^/Veh mice vs. *Shati* ^−/−^/Veh mice, F_3,39_ = 2.862, *p* < 0.05). In the three-chambered social interaction test, reduced social interaction of *Shati* ^−/−^ mice was rescued to the level of *Shati* ^+/+^ mice by GTA treatment (Fig. 2d; Strenger; *Shati* ^+/+^/Veh mice = 63.91 ± 1.62%, *Shati* ^−/−^/Veh mice = 50.06 ± 2.29%, *Shati* ^+/+^/GTA mice = 64.63 ± 1.47%, *Shati* ^−/−^/GTA mice = 63.11 ± 1.69%; *Shati* ^+/+^/Veh mice vs. *Shati* ^−/−^/Veh mice F_3,47_ = 5.551; *p* < 0.001, *Shati* ^−/−^/Veh mice vs. Shati^−/−^/GTA mice, F_3,47_ = 5.188, *p* < 0.001; *Shati* ^+/+^/GTA mice vs. *Shati* ^−/−^/GTA mice, F_3,47_ = 0.3627, n.s). Similarly, the increased time that *Shati* ^−/−^ mice spent the open arms in the elevated plus maze compared with *Shati* ^+/+^ was normalized by GTA treatment (Fig. 2e; *Shati* ^+/+^ */*Veh mice = 38.49 ± 27.7 s, *Shati* ^−/−^ */*Veh mice = 81.62 ± 6.75 s, *Shati* ^+/+^/GTA mice = 49.49 ± 3.14 s, *Shati* ^−/−^/GTA mice = 52.16 ± 3.69 s; *Shati* ^+/+^/Veh mice vs. *Shati* ^−/−^/Veh mice, F_3,47_ = 6.608, *p* < 0.001; *Shati* ^−/−^/Veh mice vs. *Shati* ^+/+^/GTA mice, F_3,47_ = 4.923, *p* < 0.001; *Shati* ^−/−^/Veh mice vs. *Shati* ^−/−^/GTA mice, F_3,47_ = 4.514, *p* < 0.001). To investigate the effect of NAA on the brain of the adult mice, we administrated NAA for the mice into the cerebral ventricle at every test day. In the social interaction test, there was no difference of the approach time of *Shati* ^−/−^/Veh mice and *Shati* ^−/−^/NAA mice (Fig. 3a; *Shati* ^+/+^ */*Veh mice = 69.04 ± 3.20%, *Shati* ^−/−^ */*Veh mice = 52.11 ± 2.84%, *Shati* ^+/+^/NAA mice = 65.28 ± 3.78%, *Shati* ^−/−^/NAA mice = 53.20 ± 3.05%; *Shati* ^+/+^/Veh mice vs. *Shati* ^−/−^/Veh mice, F_3,31_ = 5.268, *p* < 0.01; *Shati* ^+/+^/Veh mice vs. *Shati* ^−/−^/NAA mice, F_3,31_ = 4.926, *p* < 0.05; *Shati* ^−/−^/Veh mice vs. *Shati* ^+/+^/NAA mice, F_3,31_ = 4.097, *p* < 0.05). Moreover, to investigate which period is important for the affection of the behavioral deficits in *Shati* ^−/−^ mice, we only treated GTA for the mice from postnatal day 7 to 21. GTA normalized social interaction of *Shati* ^−/−^ mice as the level of *Shati* ^+/+^ mice groups, although *Shati* ^−/−^ mice showed reduced social interaction compared with *Shati* ^+/+^ mice (Fig. 3b; *Shati* ^+/+^ */*Veh mice = 61.40 ± 1.81%, *Shati* ^−/−^ */*Veh mice = 50.71 ± 1.71%, *Shati* ^+/+^/GTA mice = 60.32 ± 2.02%, *Shati* ^−/−^/GTA mice = 59.18 ± 1.40%; *Shati* ^+/+^/Veh mice vs. *Shati* ^−/−^/Veh mice, F_3,31_ = 6.573, *p* < 0.001; *Shati* ^−/−^/Veh mice vs. *Shati* ^+/+^/GTA mice, F_3,31_ = 5.905, *p* < 0.01; *Shati* ^−/−^/Veh mice vs. *Shati* ^−/−^/GTA mice, F_3,31_ = 5.199, *p* < 0.01). We also reported that the level of *BDNF* mRNA in the prefrontal cortex of *Shati* ^−/−^ mice is decreased compared with that of *Shati* ^+/+^ mice^13^. Therefore, we also measured the level of *BDNF* mRNA in *Shati* ^+/+^ and *Shati* ^−/−^ mice treated with GTA. The expression of *BDNF* mRNA in *Shati* ^−/−^ mice was significantly decreased compared with *Shati* ^+/+^ mice similar to the previous report^13^, and GTA treatment from P7 to P21 normalized the level of BDNF mRNA in *Shati* ^−/−^ mice (Fig. 3c; *Shati* ^+/+^/Veh mice = 100 ± 13.4%, *Shati* ^−/−^/Veh mice = 65.5 ± 8.3%, *Shati* ^+/+^/GTA mice = 107.3 ± 12.4%, *Shati* ^−/−^/GTA mice = 96.1 ± 12.1%; *Shati* ^+/+^/ Veh mice vs. *Shati* ^−/−^/Veh mice, F_3,31_ = 3.526, *p* < 0.05; *Shati* ^−/−^/Veh mice vs. *Shati* ^+/+^/GTA mice, F_3,31_ = 4.274, *p* < 0.01; *Shati* ^−/−^/Veh mice vs. *Shati* ^−/−^/GTA mice, F_3,31_ = 3.128, *p* < 0.05). These findings indicate that GTA treatment ameliorates the behavioral deficits and reduction of *BDNF* mRNA caused by deletion of SHATI/NAT8L in mice.

### GTA treatment from P7 to P21 affected the myelination in the brain of P21 Shati^−/−^ mice

**Figure 4:**
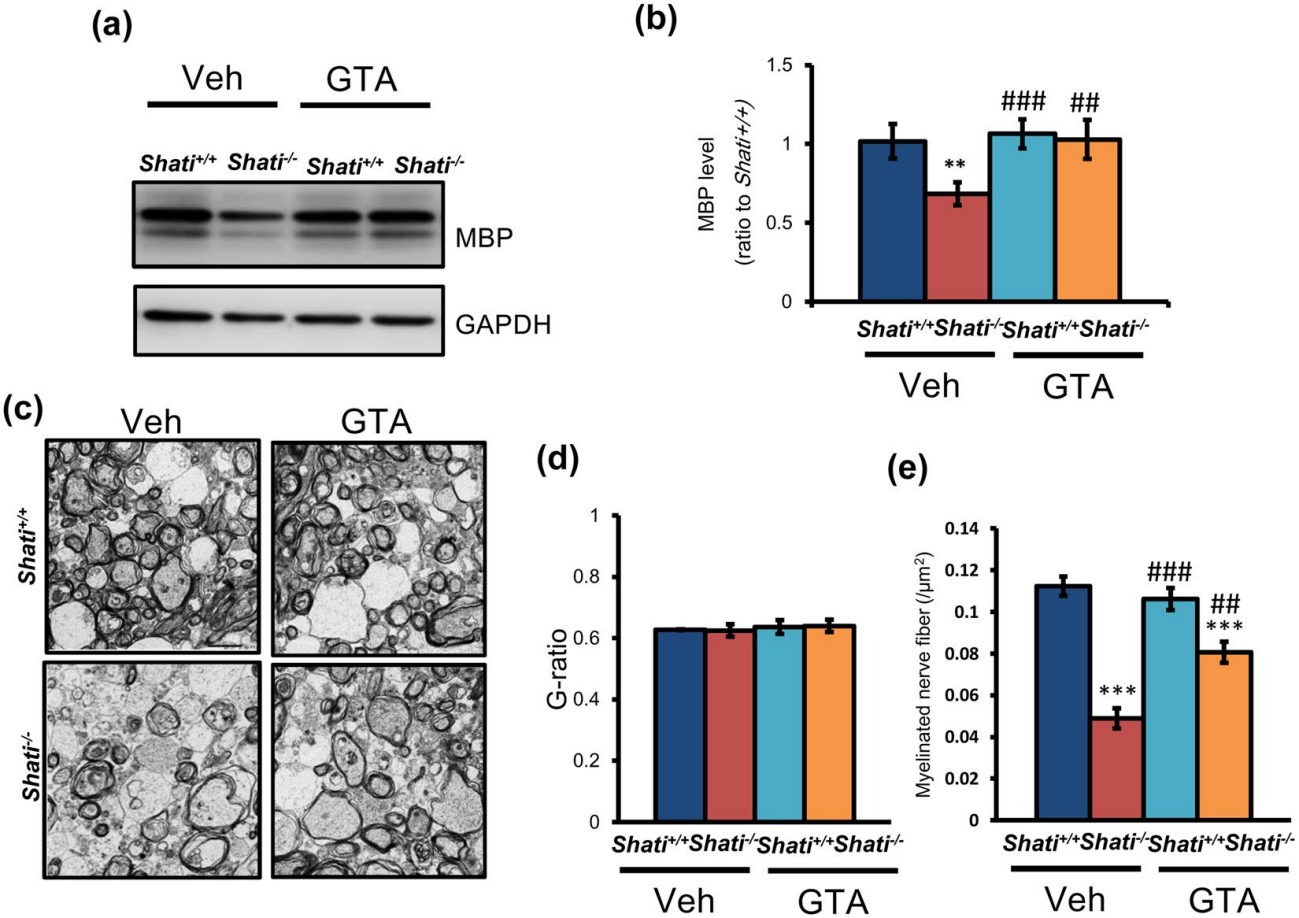
GTA treatment normalized the myelin in the brain of juvenile mice. (**a**) The expression pattern of MBP between *Shati* ^+/+^ and *Shati* ^−/−^ mice was detected by Western blot analysis. b) GTA treatment revealed the decreased expression of MBP in the brain of juvenile *Shati* ^−/−^ mice. Values represent the mean ± S.E.M. (n = 7). **p < 0.01 vs. *Shati* ^+/+^/Veh mice, ^##^ *p* < 0.01, ^###^ *p* < 0.001 vs. *Shati* ^−/−^/Veh mice (ANOVA followed by Bonferroni’s post-hoc test). Full-length blots are presented in Supplementary Fig. S4. (**c**) Electron micrographs of axons in the PFC were showed. Scale bar, 2.0 μm. (**d**) There is no difference in g ratio of the PFC between each mice group. Values represent the mean ± S.E.M. (n = 210 axons). (**e**) The number of myelinated nerve fiber in mice were indicated. GTA treatment reversed the decreased number of myelinated nerve fiber in the brain of juvenile *Shati* ^−/−^ mice. Values represent the mean ± S.E.M. (n = 45) ***p < 0.001, ^###^ *p* < 0.001, ^##^ *p* < 0.01 vs. *Shati* ^−/−^/Veh mice (ANOVA followed by Bonferroni’s post-hoc test).

**Figure 5:**
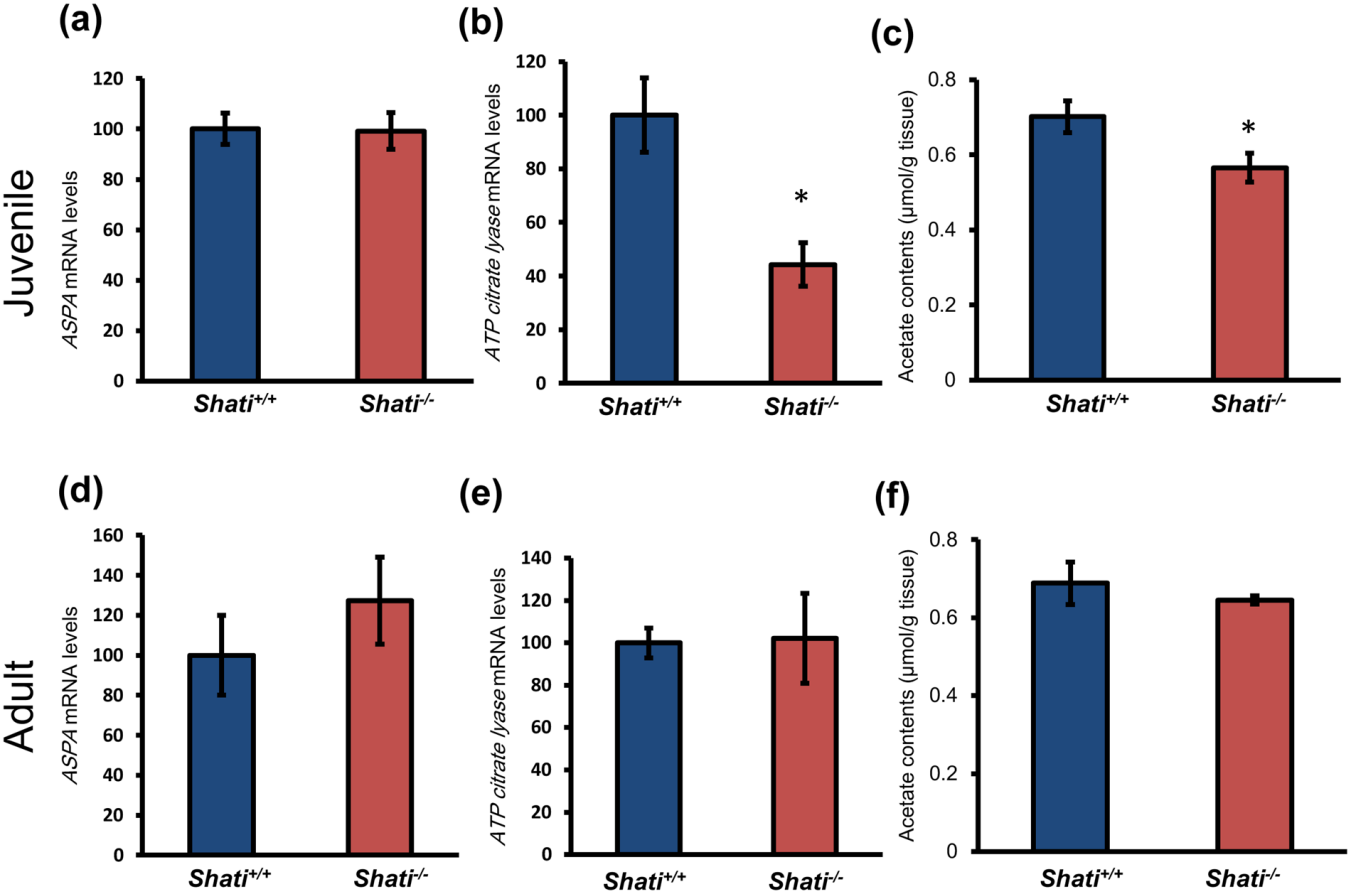
The expression of SHATI/NAT8L related genes. Real-time RT-PCR analysis of Shati/Nat8l related gens were performed. To standardize the PCR products, we used primers for 36B4 as an internal control. The expression of (**a**,**d**) *ASPA* and (**b**,**e**) *ATP citrate lyase* mRNA in juvenile and adult *Shati* ^+/+^ and *Shati* ^−/−^ mice was detected. Values represent the mean ± S.E.M. (n = 3 or 4). (**c**,**f**) The level of acetate (**c**) in the whole brain in juvenile mice and (**f**) in the frontal cortex between *Shati* ^+/+^ and *Shati* ^−/−^ mice was detected by acetate assay. Values represent the mean ± S.E.M. (n = 3 or 4) *p < 0.05 vs. *Shati* ^+/+^ mice (Student’s t test).

The expression level of MBP, one of indicators of myelin, was reduced in Juvenile of *Shati* ^−/−^ mice (Fig. 1b and d). Next, to examine whether decreased MBP in juvenile *Shati* ^−/−^ mice was ameliorated by GTA treatment, we performed Western blotting analysis to compare MBP level in the brain of *Shati* ^−/−^ mice after GTA treatment. Western blots showed that GTA treatment rescued the decreased protein level of MBP in *Shati* ^*−/−*^ mice (Fig. 4a,b; *Shati* ^+/+^/Veh mice = 1.00 ± 0.11, *Shati* ^−/−^/Veh mice = 0.67 ± 0.07, *Shati* ^+/+^/GTA mice = 1.05 ± 0.09, *Shati* ^−/−^/GTA mice = 1.01 ± 0.12; *Shati* ^+/+^/ Veh mice vs. *Shati* ^−/−^/Veh mice, F_3,27_ = 4.272, *p* < 0.01; *Shati* ^−/−^/Veh mice vs. *Shati* ^+/+^/GTA mice, F_3,27_ = 4.877, *p* < 0.001; *Shati* ^−/−^/Veh mice vs. *Shati* ^−/−^/GTA mice, F_3,27_ = 4.422, *p* < 0.01). To investigate detail the myelin condition in the brain of *Shati* ^+/+^ and *Shati* ^−/−^ mice, we used electron microscopy (Fig. 4c). There is no difference in the g-ratio between *Shati* ^+/+^ and *Shati* ^−/−^ mice (Fig. 4d; *Shati* ^+/+^/Veh mice = 0.627 ± 0.002, *Shati* ^−/−^/Veh mice = 0.625 ± 0.020, *Shati* ^+/+^/GTA mice = 0.636 ± 0.023, *Shati* ^−/−^/GTA mice = 0.640 ± 0.020; *Shati* ^+/+^/ Veh mice vs. *Shati* ^−/−^/Veh mice, vs. each groups n.s.). On the other hand, the number of myelinated nerve fiber in the prefrontal cortex of *Shati* ^−/−^ mice were significantly decreased compared with *Shati* ^+/+^ mice (Fig. 4e *Shati* ^+/+^/Veh mice = 0.112 ± 0.005 /µm^2^, *Shati* ^−/−^/Veh mice = 0.049 ± 0.005/µm^2^, *Shati* ^+/+^/GTA mice = 0.106 ± 0.005/µm^2^, *Shati* ^−/−^/GTA mice = 0.081 ± 0.005; *Shati* ^+/+^/ Veh mice vs. *Shati* ^−/−^/Veh mice, F_3,179_ = 9.063, *p* < 0.001; *Shati* ^+/+^/Veh mice vs. *Shati* ^−/−^/GTA mice, F_3,179_ = 4.526, *p* < 0.001; *Shati* ^−/−^/Veh mice vs. *Shati* ^+/+^/GTA mice, F_3,179_ = 8.176, *p* < 0.001; *Shati* ^−/−^/Veh mice vs. *Shati* ^−/−^/GTA mice, F_3,179_ = 3.639, *p* < 0.01). GTA treatment recovered the number of myelinated nerve fiber partially (Fig. 4e). Nevertheless the reduction of myelination in *Shati* ^−/−^ mice, we could not detect the TUNEL positive cell in the brain of juvenile and adult *Shati* ^−/−^ mice, respectively (Supplemental Fig. 5). Moreover, we performed Immunostaining of marker protein to confirm the number of cell. There is no difference in the number of cells between the brain of *Shati* ^+/+^ and *Shati* ^−/−^ mice (Supplemental Figs 6, 7; Olig2 positive cell, juvenile *Shati* ^+/+^ mice = 277 ± 23 cells/cm^2^, juvenile *Shati* ^−/−^ mice = 284 ± 17 cells/cm^2^ adult *Shati* ^+/+^ mice = 303 ± 24 cells/cm^2^, adult *Shati* ^−/−^ mice = 295 ± 23 cells/cm^2^; NeuN positive cell, juvenile *Shati* ^+/+^ mice = 1343 ± 66 cells/cm^2^, juvenile *Shati* ^−/−^ mice = 1318 ± 82 cells/cm^2^, adult *Shati* ^+/+^ mice = 1311 ± 127 cells/cm^2^, adult *Shati* ^−/−^ mice = 1235 ± 81 cells/cm^2^; Iba1 positive cell, juvenile *Shati* ^+/+^ mice = 233 ± 16 cells/cm^2^, juvenile *Shati* ^−/−^ mice = 232 ± 19 cells/cm^2^, adult *Shati* ^+/+^ mice = 254 ± 9 cells/cm^2^, adult *Shati* ^−/−^ mice = 245 ± 13 cells/cm^2^; GFAP positive cell, juvenile *Shati* ^+/+^ mice = 138 ± 16 cells/cm^2^, juvenile *Shati* ^−/−^ mice = 145 ± 23 cells/cm^2^, adult *Shati* ^+/+^ mice = 116 ± 16 cells/cm^2^, adult *Shati* ^−/−^ mice = 110 ± 16/cm^2^). These results indicate that dysfunction of myelination did not induce apoptosis in *Shati* ^−/−^ mice. Next, we examined the expression of SHATI/NAT8L related genes, ASPA and ATP citrate lyase. The expression of ASPA mRNA was no difference in the prefrontal cortex between juvenile and adult *Shati* ^+/+^ and *Shati* ^−/−^ mice (Fig. 5a,d; A *Shati* ^+/+^ mice = 100 ± 6.21%, *Shati* ^−/−^ mice = 99.2 ± 7.32%, t_3_ = 0.08488, n.s., D *Shati* ^+/+^ mice = 100 ± 20.00, *Shati* ^−/−^ mice = 127.4 ± 21.77%, t_3_ = 0.9269, n.s.). However, the expression of ATP citrate lyase mRNA in the prefrontal cortex of *Shati* ^−/−^ mice was significantly decreased in the juvenile, but not the adult (Fig. 5b,e B *Shati* ^+/+^ mice = 100 ± 7.13%, *Shati* ^−/−^ mice = 44.30 ± 8.14%, t_3_ = 3.461, *p* < 0.05; E *Shati* ^+/+^ mice = 100 ± 13.89%, *Shati* ^−/−^ mice = 102.15 ± 21.19%, t_3_ = 0.9632, n.s.). To assess the level of acetate in the juvenile and adult brain of *Shati* ^+/+^ and *Shati* ^−/−^ mice, we performed an acetate assay using an acetate colorimetric assay kit. The level of acetate in the juvenile prefrontal cortex was found to be decreased in *Shati* ^−/−^ mice compared with *Shati* ^+/+^ mice (Fig. 5c; *Shati* ^+/+^ mice = 0.72 ± 0.01 µmol/g tissue, *Shati* ^−/−^ mice = 0.62 ± 0.01 µmol/g tissue, t_2_ = 6.551, *p* < 0.05). On the other hand, there was no difference in the acetate level in the adult prefrontal cortex between *Shati* ^+/+^ and *Shati* ^−/−^ mice (Fig. 5F; *Shati* ^+/+^ mice = 0.69 ± 0.05, *Shati* ^−/−^ mice = 0.62 ± 0.01, t_3_ = 1.476, n.s.). These results indicate that the myelination in the brain of *Shati* ^−/−^ mice is significantly delayed compared with that of *Shati* ^+/+^ mice and GTA treatment ameliorates these impairments.

## Discussion

Although only SHATI/NAT8L was identified as *N*-acetyltransferase in brain^2,3^, NAA and NAAG are detected in the frontal cortex of mice brains. We also investigated the NAA and NAAG contents in *Shati* ^+/+^ and *Shati* ^−/−^ mice by liquid chromatography-mass spectrometry (LC-MS), which is much more sensitive than HPLC methods^9,12^. NAA was markedly decreased and NAAG was completely knocked out in *Shati* ^−/−^ mice (Sumi, Tomohiro, Hatanaka, Nitta unpublished data). Although the synthase of NAA is considered to be only SHATI/NAT8L, the result from LC-MS experiments also indicates the role of an unknown enzyme in NAA production in the brain. Further analysis is needed to elucidate the mechanisms of NAA production.

In the present study, we observed expression of *Shati/Nat8l* mRNA increases with brain development in mice (Fig. 1a). This result indicates that the function of SHATI/NAT8L is important for the brain after birth. To investigate the function of SHATI/NAT8L in the brain development, we checked the level of MBP in the juvenile and adult brain. Interestingly, deletion of SHATI/NAT8L altered the MBP level in the brain of juvenile, but not adult, mice (Fig. 1d,e). Our result is consisted with previous report that the MBP level in the adult brain of *Shati* ^−/−^ mice is no change compared with *Shati* ^+/+^ mice^23^. These results suggest that SHATI/NAT8L could be involved in the myelination in the juvenile mouse brain.

We have previously reported that a shorten immobility of *Shati* ^+/−^ mice in the forced swimming test was ameliorated by a single intracerebroventricular injection of NAA^12^. On the other hand, the same treatment of NAA could not completely normalize the behavioral deficits seen in *Shati* ^−/−^ mice, the reason for which is still unclear^12^. In the present study, we treated NAA into intracerebroventricules for adult *Shati* ^−/−^ mice repeatedly, but NAA did not improve behavioral deficit (Fig. 3b). These results indicate that treatment of NAA for adult mice dose not rescue the behavioral deficits of *Shati* ^−/−^ mice. We had also considered it is important to investigate the effect of NAA on rescuing myelination in juvenile mice. It was an important point whether treatment of NAA from jugular stage could rescue the behavioral deficits in adult *Shati* ^−/−^ mice. However, NAA could not be penetrated from periphery to brain by using intraperitoneal injection although acute and repeated NAA oral treatment did not show toxicity^24,25^. Moreover, it is technically difficult to inject to ventricles of mice in juvenile stage, since their brains are too small and brittle. NAA is metabolized to aspartate and acetate by ASPA in oligodendrocytes in the brain. Then acetate is converted to acetyl-CoA and used for lipid synthesis and myelination. Moreover, GTA is metabolized to acetate and rapidly distribute to the brain after oral administration as previous reports^21,22^. Hence, we investigated the roles of NAA in behavioral deficits using *Shati* ^−/−^ mice treated with GTA, the acetate trimester of glycerol, from P7 to P56. Interestingly, GTA treatment normalized MBP level in the brain of juvenile mice and ameliorated reduced social interaction caused by deletion of SHATI/NAT8L in adulthood (Fig. 2). Furthermore, to investigate the critical period that affects the behavioral deficits in *Shati* ^−/−^ mice, we treated GTA for mice from P7 to P21 until weaning. GTA treatments improve reduced social interaction of adult *Shati* ^−/−^ mice. Furthermore, the level of acetate in the juvenile prefrontal cortex was found to be decreased in *Shati* ^−/−^ mice compared with *Shati* ^+/+^ mice (Fig. 5c), suggesting that the presence of acetate in the juvenile period is important for social behavior.

There are several reports that impaired or delayed myelination in the prefrontal cortex induces reduced social interaction in adult mice^18,19^. Also absence of NAA and NAAG is involved in delayed myelination in patients with hypoacetylaspartia^26^. On the other hand, it was reported that BDNF signaling in the developmental brain is involved myelination^27,28^. Previously, it was reported that the levels of *BDNF* mRNA in the prefrontal cortex were decreased in *Shati* ^−/−^ mice^13^, GTA treatment for *Shati* ^−/−^ mice normalized the decrease of *BDNF* mRNA and MBP level (Figs 3c, 4a,b). Moreover, by using electron microscopy analysis, myelinated nerve filers of *Shati* ^−/−^ mice were decrease compared with that of *Shati* ^+/+^ mice although g- ratio of each groups were not changed. (Fig. 4c,d,e). As shown in Supplemental Figs 5–7, the dysfunction of myelination did not induce apoptosis in *Shati* ^−/−^ mice. Hence, these results show possibility that the recovery of behavior deficits and delayed myelination in *Shati* ^−/−^ mice by GTA treatment is associated with normalization of *BDNF* mRNA level. There is no report that acetate or GTA could directly affect expression of BDNF. Therefore, we assume that the normalization of BDNF mRNA level in *Shati* ^−/−^ mice treated with GTA might indicate amelioration of neuronal activity due to impairment of myelination in the jugular stage of the *Shati* ^−/−^ mice. BDNF mRNA expression is regulated neuronal activity^29^.

We have previously reported that SHATI/NAT8L is associated with neurite elongation and the ATP synthetic pathway via NAA synthesis^14^. SHATI/NAT8L is expressed in the mitochondria of neuronal cells, and NAA synthesized by SHATI/NAT8L is associated with the tricarboxylic acid cycle related to metabolism in neurons^14^. Further, NAA is metabolized to acetate and aspartate in the oligodendrocytes. Hence, the ameliorative effect of acetate derived from GTA on the behavioral deficits is hypothesized that it acts directly at oligodendrocytes. In the present study, we checked the expression of *ASPA* and *ATP citrate lyase* mRNA and acetate contents to investigate the effect on utilization of NAA in oligodendrocyte (Fig. 5a–f). Surprisingly, the levels of *ATP citrate lyase* mRNA and acetate contents were decreased in the PFC of juvenile, but not adult *Shati* ^−/−^ mice. These results suggested that utilization ability of NAA was decreased in the oligodendrocytes of *Shati* ^−/−^ mice. The reduced levels of acetate in the brain of *Shati* ^−/−^ mice are consisted with previous report that knock-down of the NAA-cleaving enzyme reduces acetate levels in adipocytes^30^. On the other hand, it was reported that the levels of *ATP citrate lyase* mRNA is increased in the adipocytes of *Shati* ^−/−^ mice and this report is inconsistent with our results^31^. The reasons of discrepancy between *ATP citrate lyase* mRNA in the brain and adipocytes of *Shati* ^−/−^ mice is unclear at the present. We estimate that the differences between the organs cause the result, because ASPA expressed in the adipocytes, but not the neurons. Further study is needed to clarify the detail mechanism of this discrepancy.

The findings of the current study and those of previous studies show that deletion of SHATI/NAT8L alters MBP level in the brain of juvenile, but not adult mice, suggesting that SHATI/NAT8L is involved in myelination via its role in NAA synthesis. Furthermore, *Shati* ^−/−^ mice showed several behavioral deficits, and these deficits were ameliorated by GTA treatment during the juvenile stage, suggesting that the behavioral deficits occurred due to decreased acetate. These findings suggest that SHATI/NAT8L is involved in myelination in the juvenile mouse brain via supplementation of acetate derived from NAA. It is well known that defects in NAA metabolism result in impaired postnatal myelination, most notably in Canavan disease^23^, SHATI/NAT8L might be involved in brain development, especially, in myelination, and may be therapeutic targets for developmental disorders. The number of patients with developing disorders is much more than Canavan disease. The pharmaceutical therapy is required for developing disorders, but we have no means at the present. Then our results will contribute the development of the medical tools for developing disorders. The absence of NAA and NAAG is involved in delayed myelination in humans. Therefore, it is possible that these molecules participate in other developmental disorders^26^. We expect that SHATI/NAT8L will become a novel therapeutic target for the treatment of cryptogenic developmental disorders.

## Materials and Methods

### Animals

We have previously described the generation of *Shati* ^−/−^ mice^18^. Animals were housed in a room with a 12 h light/dark cycle (light cycle starting at 8:00 AM.). Food and water were available ad libitum. All experiments followed the National Institute of Health Guidelines for the Care and Use of Laboratory Animals and were approved by the committee for Animal Experiments of the University of Toyama (A2015PHA-23, G2015PHA-15).

### Administration of glyceryltriacetate (GTA) and N-acetylaspatate (NAA)

Glyceryltriacetate (GTA; Wako, Osaka, Japan) treatment was performed as previously described^21,22^. GTA was treated orally to *Shati* ^+/+^ and *Shati* ^−/−^ pups from day 7 after birth until day 14 at a dose of 4.2 g/kg. 5.8 g/kg GTA was administered from day 15 to day 21. After weaning (after day 22), the pups received GTA in their water (5% GTA by weight). Intracerebroventricular injection of NAA was performed as previously described^12^. Briefly, a microsyringe with a 28-gauge stainless-stell needle (3 mm in length) was used for the microinjection. The mice were lightly anesthetized and the needle was implanted into the lateral ventricle (AP −0.6 mm, ML +1.0 mm, DV −2.5 mm). NAA was solubilized in Saline to obtain a concentration of 20 µg/µL. The i.c.v. injection volume was 3 µL, 30 min before each Three-chambered social interaction test trail, and the injection speed was 20 s.

### Schedule of behavioral tests and sampling for brain tissues

All behavioral tests were performed from the age of 8-9 weeks old in the following order so as to reduce the stress on the mice; locomotor activity, Y-maze test, three-chambered social interaction test, and elevated plus maze test. After the behavioral tests, brain samples were collected and used for Western blotting or acetate assay. The brains used for the experiments with electron microscopy were separately prepared. Behavioral tests were finished during the ages of 9–10 weeks old, and sampling was performed when the mice became 10 weeks old (Fig. 2a).

### Quantitative RT-PCR

Quantitative RT-PCR was performed as previously described^14^. The *Shati/Nat8l* primers used for real-time PCR were as follows: 5′-GTGATTCTGGCCTACCTGGA-3′ (forward) and 5′-CCACTGTGTTGTCCTCCTCA-3′ (reverse). The other primers were as follows: 5′-GCAAACATGTCTATGAGGGTTCG-3′ (BDNF forward), 5′-ACTCGCTAATACTGTCACACACG-3′ (BDNF reverse), 5′-GAAGCTGACCTTGCTGAACC-3′ (ATP citrate lyase forward), 5′-CCGTAATTCGCCAGTTCATT-3′ (ATP citrate lyase reverse), 5′-CATTGAGCATCCTT-3′ (ASPA forward), 5′-TGAGGCTGAGGACCAACTTC-3′ (ASPA reverse) 36B4 transcript was used as the internal control. The amount of 36B4 transcript was quantified using the forward primer 5′-ACCCTGAAGTGCTCGACATC-3′ and the reverse primer 5′-AGGAAGGCCTTGACCTTTTC-3′.

### Immunostaining of mice brains

Immunostaining was performed as previously described^14^. Sections were fixed with 4% paraformaldehyde in 20 mM Tris-HCl (pH 7.4) containing 150 mM NaCl, 3 mM KCl, and 0.1% Tween 20 (TBS-T) for 20 min, washed with TBS-T, and then incubated with 0.25% Triton X-100 in TBS-T for 15 min. The sections were treated with 10 mM citrate solution (pH 6.0) for antigen retrieval at 95 °C for 15 min, washed with TBS-T, and then blocked in 10% goat serum (Sigma-Aldrich, St. Louis, MO) in TBS-T for 1 h. Sections were incubated with primary antibody (MBP, 1:500 BioLegend, San Diego, CA; Olig2, 1:500 Abcam, cambridge UK; NeuN, 1:500 MBL, Nagoya, JAPAN; GFAP, 1:500 Cell Signaling Technology, Beverly MA; Iba1, 1:500 Wako, Japan) with 10% goat serum in TBS-T at 4 °C overnight, washed with TBS-T, and then incubated with CF^TM^ 594 goat anti-rabbit IgG(H + L) (1:1000 Biotium, Hayward, CA) and CF^TM^ 488 goat anti-mouse IgG (H + L) (1:1000 Biotium) at room temperature for 2 h. After being washed, the sections were mounted using Fluoromount (Diagnostic BioSystems, Pleasanton, CA).

### Western blotting

Brains were isolated and cut into 1 mm-thick sections. The prefrontal cortex was isolated from the brain section and fractured in RIPA buffer (50 mM Tris-HCl pH 7.5, 152 mM NaCl, 5 mM EDTA, 1% TritonX-100, 0.5% sodium deoxy cholate, 1 mM PMSF, 2% protease inhibitor cocktail, and 1% phosphatase inhibitor cocktail). After centrifugation, the supernatant was collected in a fresh tube and the protein concentration was measured (BCA kit, Wako). Equal amounts of protein from each sample were mixed with loading buffer (50 mM Tris-HCl pH 7.5, 5% 2-mercaptoethanol, 2% sodium dodecyl sulfate (SDS), 5% sucrose, and 0.005% bromophenol blue) and then denatured at 100 °C. The protein extracts were subjected to SDS-polyacrylamide gel electrophoresis (SDS-PAGE) (10% acryl amide gel) in electrophoresis running buffer and electrophoresed for 1 h at room temperature at 0.2 mA, and then transferred onto a membrane (Millipore, Darmstadt, Germany) for 1 h at 100 V. The membrane was blocked with 5% skim milk powder in TBS-T for 1 h. After washing by TBS-T, the membrane was incubated with primary antibodies (MBP, BioLegend, 1:1000; GAPDH, MBL, Nagoya, JAPAN) overnight. After extensive rinsing, the membrane was incubated with a secondary antibody (Anti-Mouse IgG HRP-Linked Fragment, Cell Signaling Technology, Danvers, MA) for 1 h at room temperature. The corresponding bands were detected using an ECL-plus Western Blotting Detection System (GE Healthcare, Little Chalfont, UK). Densitometry of western blot data was performed using image j software. To account for several isoforms of MBP, protein bands from 10 kDa to 25 kDa were used^17^.

### Measurement of locomotor activity

Measurement of locomotor activity was performed as previously described^9^. Mice were placed individually in a transparent acrylic cage with a black frosted Plexiglas floor (45 × 25 × 40 cm), and locomotor activity was measured every 5 min for 60 min using digital counters with infrared sensors (Scanet MV-40; MELQEST, Toyama, Japan).

### Y-maze test

Measurement of spontaneous alternation behavior was performed as previously described^13^. The percentage alternation was calculated using the following formula: (number of alternations)/(total number of arm entries-2) × 100 (%).

### Three-chambered social interaction test

The social interaction test was performed using a three-chambered plastic box (60 × 40 × 22 cm, MELQEST), as described in a previous report^32^. An unfamiliar C57BL/6J male (Stranger) that had no contact with the subject mice were placed in one side of the chamber, and an object was placed on the other side. The stranger mouse and the object were enclosed in a small, round wire cage, which allowed olfactory, visual, auditory, and tactile contact, but did not allow for deep contact. The subject mouse was first placed in the middle chamber and allowed to explore the entire social test box for a 10 min session. Measurement of the interaction time was taken from the amount of time spent around the wire cage.

### Elevated plus maze test

The elevated plus maze test was performed as previously described^13^. In brief, this maze is comprised of two open arms (25 × 5 × 5 cm), two closed arms (25 × 5 × 27 cm), and a home platform (5 × 5 cm). It was elevated to a height of 55 cm above the floor. The time spent in open arm was measured.

### Electron microscopy

Electron microscopy was performed as previously described^11^. In brief, *Shati* ^+/+^ and *Shati* ^−/−^ mice at postnatal day 21 were anesthetized and perfused intracardially with 2.0% glutaraldehyde in 0.1 M cacodylate buffer pH 7.4 for 15 min. The brains were removed and dissected 1–2 mm thick section which included the prefrontal cortex. The sections were left in fixative overnight at 4 °C then washed in 0.1 M cacodylate buffer, dehydrated with graded ethanols and infiltrated with propylene oxide. After infiltration of propylene oxide, the section was oriented and embedded with epoxy resin. Sections (1 µm) of the specimen block were cut on Ultracut micotome (Leica), stained with 0.5% toluidine blue in 1% sodium borate in water and prefrontal cortex was then identified by light microscopy and areas were selected for thin sectioning. Thin sections (100 nm) were collected on copper grids, stained with uranyl acetate and lead citrate. The samples were viewed at electron microscope.

### Acetate assay

Acetate assay were performed using an acetate colorimetric assay kit (BioVision, Milpitas, CA), following the manufacturer’s instructions.

### TUNEL staining

TUNEL staining was performed using *In situ* Apoptosis Detection Kit (Takara, Kusatsu, JAPAN), following the manufacturer’s instructions.

### Statistical Analyses

All data were expressed as the mean ± standard error of the mean (S.E.M.). Statistical differences between two groups were determined by Student’s t-test. Statistical differences among values for individual groups were determined by analysis of variance (ANOVA), followed by Bonferroni’s post-hoc test when *F* ratios were significant (Prism version 5).

## Electronic supplementary material

Supplementary information
