# Supplementary material for: Behavioral impairment in SHATI/NAT8L knockout mice via dysfunction of myelination development
Source: Sci Rep. 2017 Dec 4;7:16872. doi: 10.1038/s41598-017-17151-1 (PMC5715020; doi:10.1038/s41598-017-17151-1)
Supplement: Supplementary file 1 — Supplementary information [file 41598_2017_17151_MOESM1_ESM.pdf]

Supplementary information

Title: Behavioral impairment in SHATI/NAT8L knockout mice *via* dysfunction of myelination development

Authors: Kazuyuki Sum, Kyosuke Uno, Hiroshi Noike, Takenori Tomohiro, Yasumaru Hatanaka, Yoko Furukawa-Hibi, Toshitaka Nabeshima[4], Yoshiaki Miyamoto, Atsumi Nitta

Figure S1. Deletion of SHATI/NAT8L induced several behavioral deficits in mice.

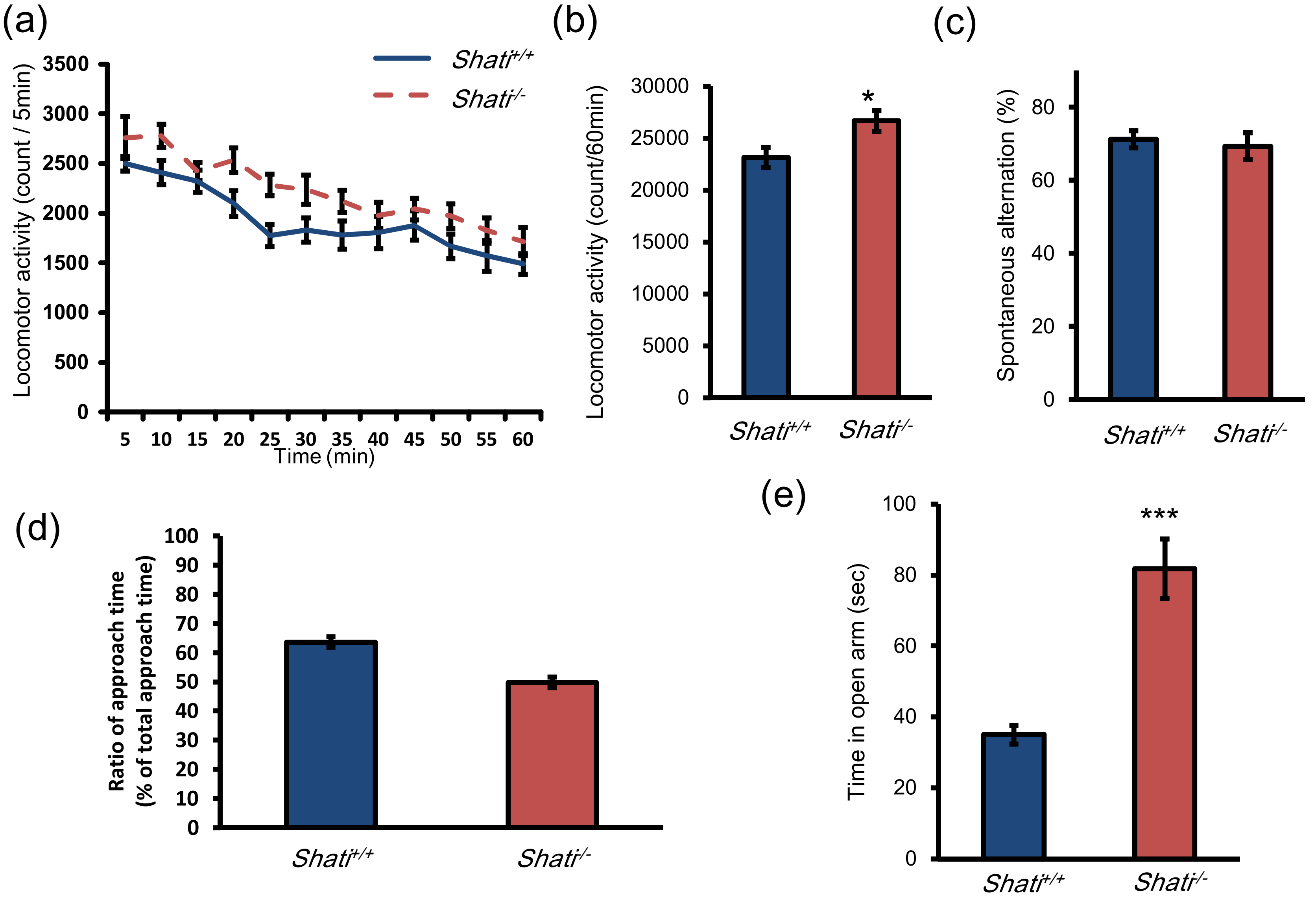

Figure S2. The full-length blots of the cropped pictures shown in Fig.1d

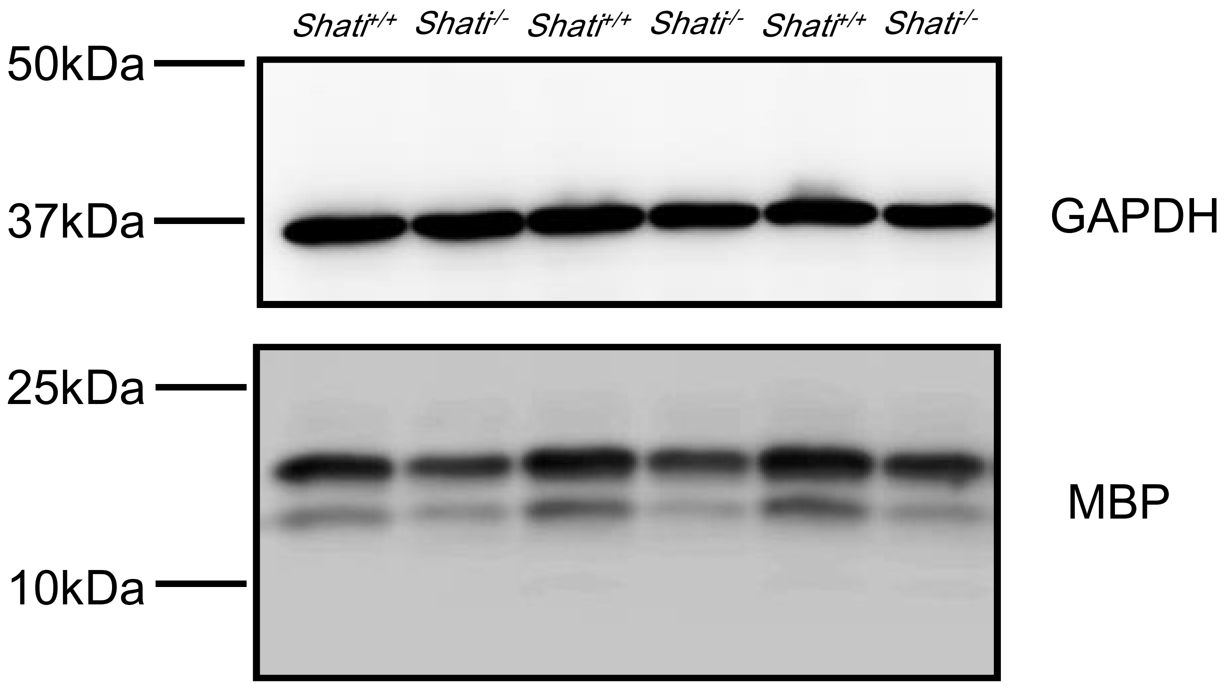

Figure S3. The full-length blots of the cropped pictures shown in Fig.1e

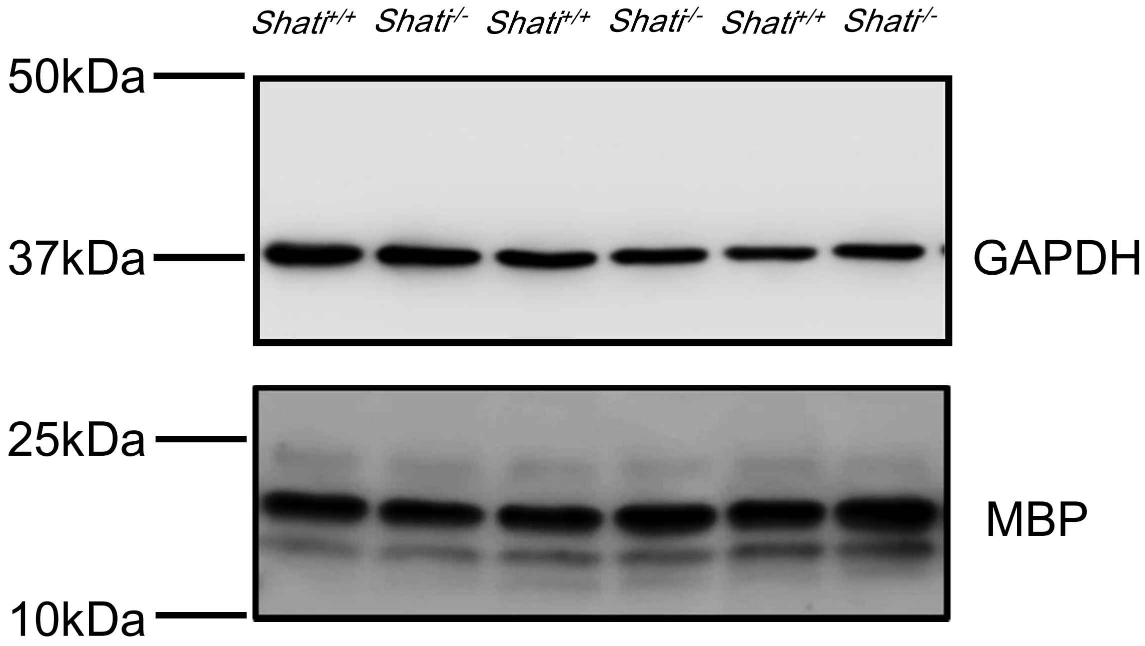

Figure S4. The full-length blots of the cropped pictures shown in Fig.4a

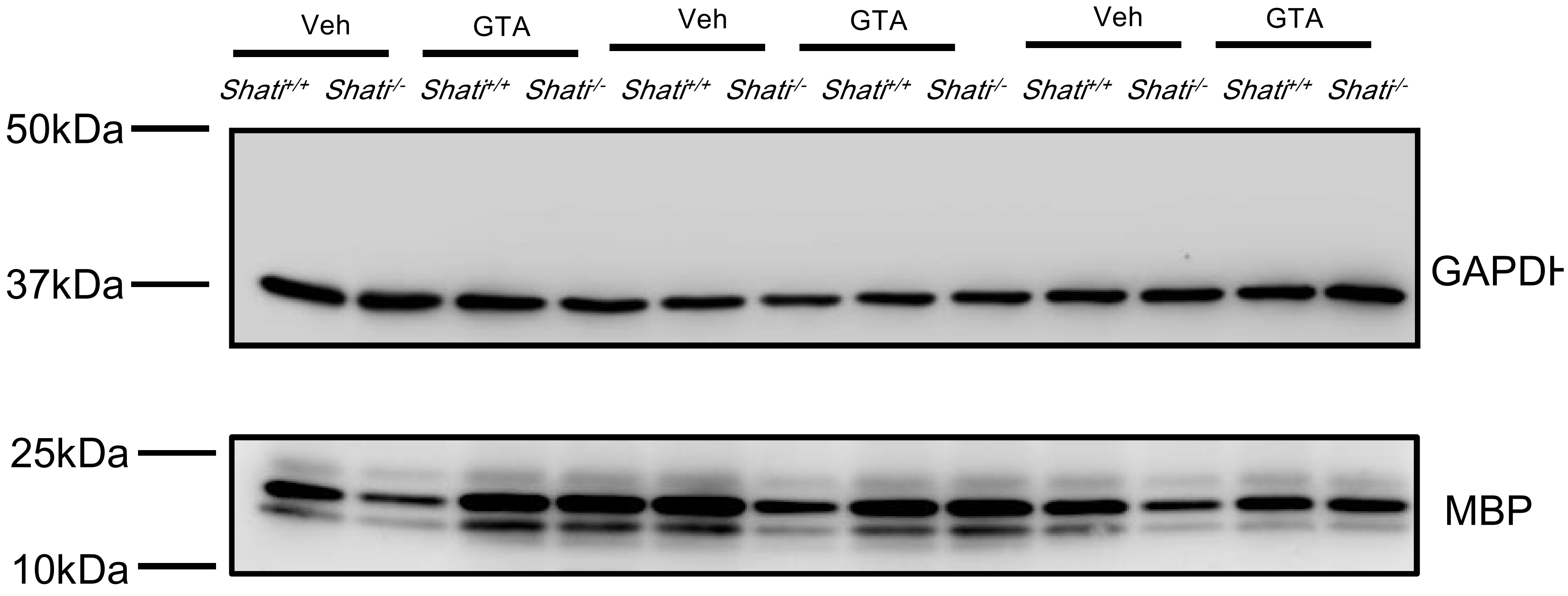

Figure S5. TUNEL staining of the brain

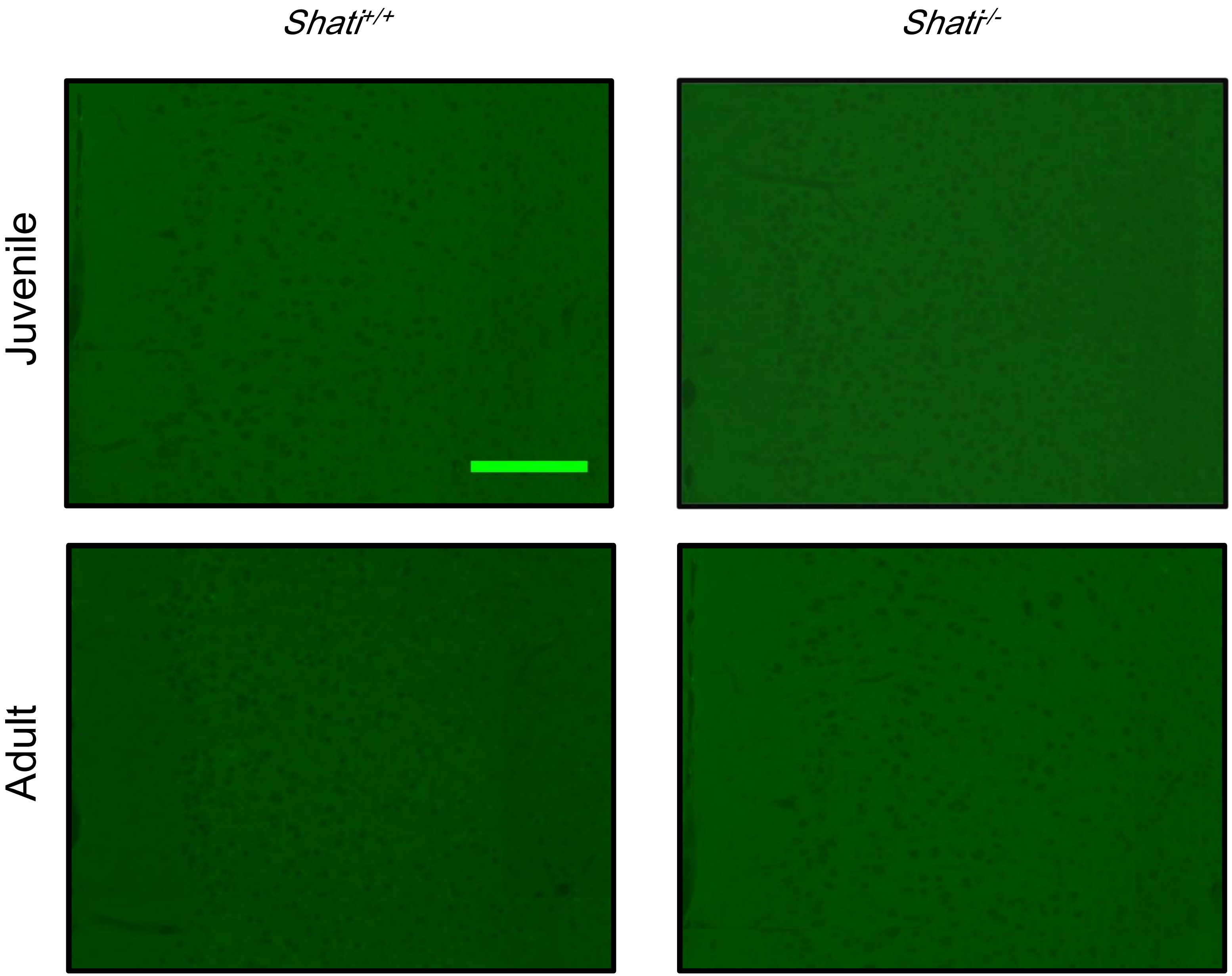

Figure S6. Immunostaining of the brain

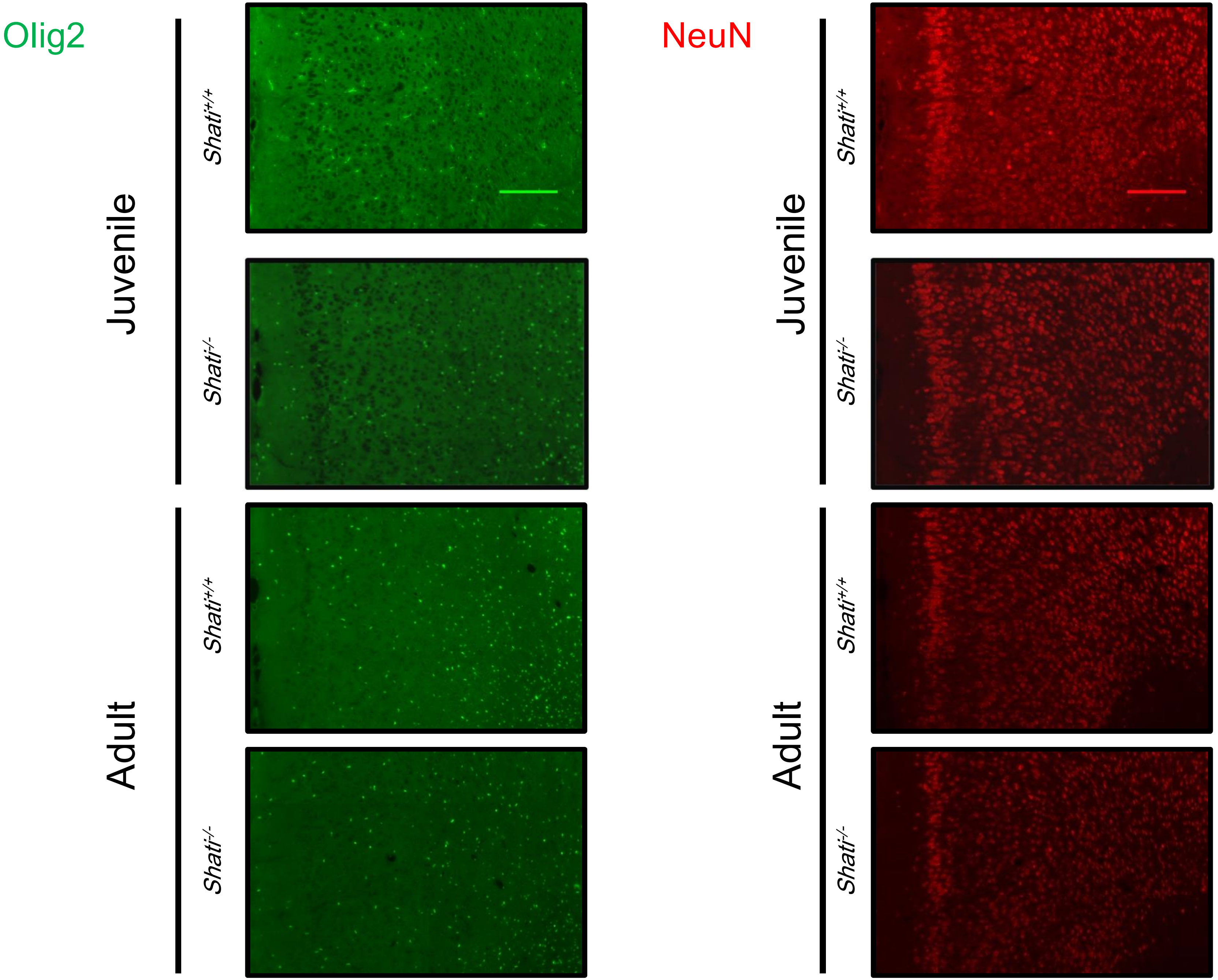

Figure S7. Immunostaining of the brain

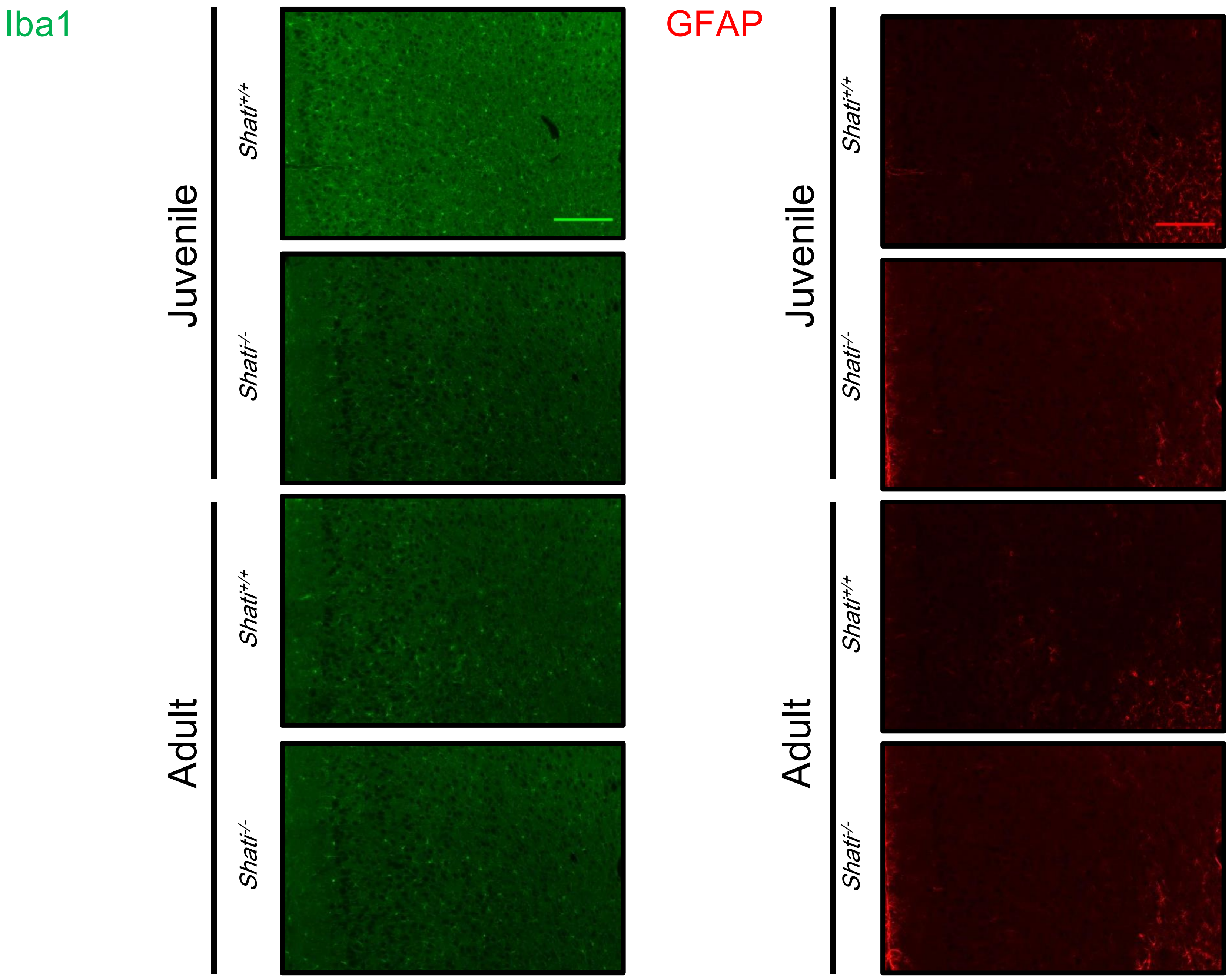

Supplementary Figure S1. Deletion of SHATI/NAT8L induced several behavioral deficits in mice.
(a,b) *Shati*$^{-/-}$ mice showed hyper activity in a novel environment compared with *Shati*$^{+/+}$ mice. Values represent the mean ± SEM. (n = 15). *p < 0.05 vs. *Shati*$^{+/+}$ mice (Student's t test) (c) No difference in spontaneous alternation behavior in the Y-maze test between *Shati*$^{+/+}$ and *Shati*$^{-/-}$ mice. Values represent the mean ± SEM. (n = 9). (d) In three-chambered social interaction test, a novel object was placed in a wire cage in the chamber on one side, and a stranger mouse (C57BL/6J) was placed in a wire cage in the chamber on the other side. *Shati*$^{-/-}$ mice were more interested to a novel object, but not to a stranger mouse compared with *Shati*$^{+/+}$ mice. Values represent the mean ± S.E.M. (n = 9). ***p < 0.001 vs. *Shati*$^{+/+}$ mice (Student's t test). (e) *Shati*$^{-/-}$ mice spent long duration in the open arms of the elevated plus-maze test compared with Shati+/+ mice. Values represent the mean ± S.E.M. (n = 9) ***p < 0.001 vs. *Shati*$^{+/+}$ mice (Student's t test)

Supplementary Figure S2. The full-length blots of the cropped pictures shown in Fig.1d

Supplementary Figure S3. The full-length blots of the cropped pictures shown in Fig.1e

Supplementary Figure S4. The full-length blots of the cropped pictures shown in Fig.4a

Supplementary Figure S5. TUNEL staining of the brain
Apoptosis cell in juvenile (3 weeks old) and adult (10 weeks old) *Shati*$^{+/+}$ and *Shati*$^{-/-}$ mice was detected by TUNEL staining. Scale bars in the figure = 200 μm

Supplementary Figure S6. Immunostaining of the brain
The expression pattern of Olig2 and NeuN in juvenile (3 weeks old) and adult (10 weeks old) *Shati*$^{+/+}$ and *Shati*$^{-/-}$ mice was detected by immunohistochemistry analysis. Scale bars in the figure = 100 μm

Supplementary Figure S7. Immunostaining of the brain
The expression pattern of Iba1 and GFAP in juvenile (3 weeks old) and adult (10 weeks old) *Shati*$^{+/+}$ and *Shati*$^{-/-}$ mice was detected by immunohistochemistry analysis. Scale bars in the figure = 100 μm
